# Monitoring of carbon-water fluxes at Eurasian meteorological stations using random forest and remote sensing

**DOI:** 10.1038/s41597-023-02473-9

**Published:** 2023-09-07

**Authors:** Mingjuan Xie, Xiaofei Ma, Yuangang Wang, Chaofan Li, Haiyang Shi, Xiuliang Yuan, Olaf Hellwich, Chunbo Chen, Wenqiang Zhang, Chen Zhang, Qing Ling, Ruixiang Gao, Yu Zhang, Friday Uchenna Ochege, Amaury Frankl, Philippe De Maeyer, Nina Buchmann, Iris Feigenwinter, Jørgen E. Olesen, Radoslaw Juszczak, Adrien Jacotot, Aino Korrensalo, Andrea Pitacco, Andrej Varlagin, Ankit Shekhar, Annalea Lohila, Arnaud Carrara, Aurore Brut, Bart Kruijt, Benjamin Loubet, Bernard Heinesch, Bogdan Chojnicki, Carole Helfter, Caroline Vincke, Changliang Shao, Christian Bernhofer, Christian Brümmer, Christian Wille, Eeva-Stiina Tuittila, Eiko Nemitz, Franco Meggio, Gang Dong, Gary Lanigan, Georg Niedrist, Georg Wohlfahrt, Guoyi Zhou, Ignacio Goded, Thomas Gruenwald, Janusz Olejnik, Joachim Jansen, Johan Neirynck, Juha-Pekka Tuovinen, Junhui Zhang, Katja Klumpp, Kim Pilegaard, Ladislav Šigut, Leif Klemedtsson, Luca Tezza, Lukas Hörtnagl, Marek Urbaniak, Marilyn Roland, Marius Schmidt, Mark A. Sutton, Markus Hehn, Matthew Saunders, Matthias Mauder, Mika Aurela, Mika Korkiakoski, Mingyuan Du, Nadia Vendrame, Natalia Kowalska, Paul G. Leahy, Pavel Alekseychik, Peili Shi, Per Weslien, Shiping Chen, Silvano Fares, Thomas Friborg, Tiphaine Tallec, Tomomichi Kato, Torsten Sachs, Trofim Maximov, Umberto Morra di Cella, Uta Moderow, Yingnian Li, Yongtao He, Yoshiko Kosugi, Geping Luo

**Affiliations:** 1 State Key Laboratory of Desert and Oasis Ecology, Xinjiang Institute of Ecology and Geography, Chinese Academy of Sciences, Urumqi, Xinjiang 830011 China; 2 Department of Geography, Ghent University, Ghent, 9000 Belgium; 3 College of Resources and Environment, University of Chinese Academy of Sciences, Beijing, 100049 China; 4 Sino-Belgian Joint Laboratory for Geo-Information, Urumqi, China; 5 Sino-Belgian Joint Laboratory for Geo-Information, Ghent, Belgium; 6 School of Geographical Sciences, Nanjing University of Information Science and Technology, Nanjing, 210044 China; 7 School of Earth Sciences and Engineering, Hohai University, Nanjing, 211100 China; 8 Department of Computer Vision & Remote Sensing, Technische Universität Berlin, 10587 Berlin, Germany; 9 Department of Geography and Environmental Management, University of Port Harcourt, PMB 5323 Choba, East-West, Port Harcourt, Nigeria; 10 Department of Environmental Systems Science, Institute of Agricultural Sciences, ETH Zürich, 8092 Zürich, Switzerland; 11 Department of Agroecology, Aarhus University, Tjele, Denmark; 12 Laboratory of Bioclimatology, Department of Ecology and Environmental Protection, Faculty of Environmental and Mechanical Engineering, Poznan University of Life Sciences, Piatkowska 94, 60-649 Poznan, Poland; 13 Sol, Agro et hydrosystèmes, Spatialisation (SAS), UMR 1069, INRAE, Institut Agro, 35000 Rennes, France; 14 Department of Environmental and Biological Sciences, University of Eastern Finland, Joensuu campus, P.O Box 111, Joensuu, FI-80101 Finland; 15 Natural Resources Institute Finland, Joensuu, Yliopistokatu 6, FI-80130 Joensuu, Finland; 16 University of Padova - DAFNAE, Viale dell’Università 16, I-35020 Padova, Legnaro (PD) Italy; 17 A.N Severtsov Institute of Ecology and Evolution, Russian Academy of Sciences, 119071, Leninsky pr.33, Moscow, Russia; 18 Climate System Research, Finnish Meteorological Institute, P.O Box 503, FI-00101 Helsinki, Finland; 19 University of Helsinki, Institute for Atmospheric and Earth System Research (INAR)/Physics, Faculty of Science, Helsinki, Finland; 20 Fundación CEAM, Parque Tecnológico, C/Charles R. Darwin, 14, Paterna, 46980 Spain; 21 CESBIO, Université de Toulouse, CNES/CNRS/INRAE/IRD/UPS, Toulouse, France; 22 Wageningen Univertsity, Water Systems and Global change group, PO bx 47, 7700AA Wageningen, Netherlands; 23 ECOSYS, INRAE, AgroParisTech, Université Paris-Saclay, 22 place de l’agronomie, 91120 Palaiseau, France; 24 Terra Teaching and Research Center, University of Liège – Gembloux Agro-Bio Tech, 5030 Gembloux, Belgium; 25 UK Centre for Ecology & Hydrology (UKCEH), Bush Estate, Penicuik, EH26 0QB UK; 26 Earth and Life Institute, Université Catholique de Louvain, 1348 Louvain-la-Neuve, Belgium; 27 National Hulunber Grassland Ecosystem Observation and Research Station & Institute of Agricultural Resources and Regional Planning, Chinese Academy of Agricultural Sciences, Beijing, 100081 China; 28 Institute of Hydrology and Meteorology, TUD Dresden University of Technology, Pienner Str. 23, 01737 Tharandt, Germany; 29 Thünen Institute of Climate-Smart Agriculture, 38116 Braunschweig, Germany; 30 GFZ German Research Centre for Geosciences, Telegrafenberg, 14473 Potsdam, Germany; 31 School of Forest Sciences, University of Eastern Finland, P.O Box 111, FIN-80100 Joensuu, Finland; 32 School of Life Science, Shanxi University, Taiyuan, 030006 China; 33 Teagasc, Johnstown Castle, Wexford, Ireland; 34 Eurac research, Institute for Alpine Environment, Viale Druso 1, 39100 Bolzano, Italy; 35 Institut für Ökologie, Universität Innsbruck, Innrain 52, 6020 Innsbruck, Austria; 36 Institute of Ecology and School of Applied Meteorology, Nanjing University of Information Science & Technology, Nanjing, 210044 China; 37 European Commission, Joint Research Centre (JRC), Ispra, Italy; 38 Laboratory of Meteorology, Department of Construction and Geoengineering, Faculty of Environmental and Mechanical Engineering, Poznan University of Life Sciences, Piatkowska 94, 60-649 Poznan, Poland; 39 Department of Ecology and Genetics/Limnology, Uppsala University, Norbyvägen 18 D, 752 36 Uppsala, Sweden; 40 Research Institute for Nature and Forest, Geraardsbergen, 9500 Belgium; 41 School of life sciences, Qufu Normal University, 57 Jingxuan West Road, Qufu, 273165 Shandong China; 42 Grassland Ecosystem Research, INRAE, VetAgro-Sup, University of Clermont Auvergne, 5 Chemin de Beaulieu, 63000 Clermont Ferrand, France; 43 Department of Environmental Engineering, Technical University of Denmark (DTU), Kgs, Lyngby, 2800 Denmark; 44 Department of Matter and Energy Fluxes, Global Change Research Institute CAS, Bělidla 986/4a, CZ-603 00 Brno, Czech Republic; 45 Departement of Earth Sciences, Gothenburg University, Guldhedsgatan 5A, Po.Box 460, SE 405 30 Gothenburg, Sweden; 46 Department of Biology, University of Antwerp, Wilrijk, 2610 Belgium; 47 Agrosphere Institute IBG-3, Forschungszentrum Jülich, Jülich, 52425 Germany; 48 School of Natural Sciences, Botany Discipline, Trinity College Dublin, D2 Dublin, Ireland; 49 National Agriculture and Food Research Organization, Tsukuba, Ibaraki 305-8517 Japan; 50 Center Agriculture Food Environment, University of Trento, Via Edmund Mach 1, I-38010 Trento, San Michele all’Adige (TN) Italy; 51 School of Engineering & Architecture, University College Cork, College Road, Cork, T12 K8AF Republic of Ireland; 52 Natural Resources Institute Finland, Bioeconomy and environment, 00790 Helsinki, Finland; 53 Lhasa Station, Key Laboratory of Ecosystem Network Observation and Modeling, Institute of Geographic Sciences and Natural Resources Research, Chinese Academy of Sciences, Beijing, 100101 China; 54 State Key Laboratory of Vegetation and Environmental Change, Institute of Botany, Chinese Academy of Sciences, Beijing, 100093 China; 55 National Research Council of Italy, Institute for Agriculture and Forestry Systems in the Mediterranean, Portici, Naples, Italy; 56 Department of Geosciences and Natural Resource Management, University of Copenhagen, Oester Voldgade 10, 1350 Copenhagen K, Denmark; 57 Research Faculty of Agriculture, Hokkaido University, Sapporo, Hokkaido 060-8589 Japan; 58 Institute for Biological Problems of Cryolithozone, Siberian Branch of the Russian Academy of Sciences, Yakutsk, Russia; 59 Climate Change Dept., Environmental Protection Agency of Aosta Valley, Saint-Christophe, I-11020 Italy; 60 Northwest Institute of Plateau Biology, Chinese Academy of Sciences, Qinghai, Xining 810008 China; 61 Laboratory of Forest Hydrology, Graduate School of Agriculture, Kyoto University, 606-8502 Kyoto, Japan; 62 The National Key Laboratory of Ecological Security and Sustainable Development in Arid Region (proposed), Chinese Academy of Sciences, Urumqi, China

**Keywords:** Ecology, Hydrology

## Abstract

Simulating the carbon-water fluxes at more widely distributed meteorological stations based on the sparsely and unevenly distributed eddy covariance flux stations is needed to accurately understand the carbon-water cycle of terrestrial ecosystems. We established a new framework consisting of machine learning, determination coefficient (R^2^), Euclidean distance, and remote sensing (RS), to simulate the daily net ecosystem carbon dioxide exchange (NEE) and water flux (WF) of the Eurasian meteorological stations using a random forest model or/and RS. The daily NEE and WF datasets with RS-based information (NEE-RS and WF-RS) for 3774 and 4427 meteorological stations during 2002–2020 were produced, respectively. And the daily NEE and WF datasets without RS-based information (NEE-WRS and WF-WRS) for 4667 and 6763 meteorological stations during 1983–2018 were generated, respectively. For each meteorological station, the carbon-water fluxes meet accuracy requirements and have quasi-observational properties. These four carbon-water flux datasets have great potential to improve the assessments of the ecosystem carbon-water dynamics.

## Background & Summary

The eddy-covariance flux stations provide reliable ecosystem-scale measurements of the carbon and energy fluxes at a high temporal resolution^1^. They have become crucial tools to generate observation datasets to verify and benchmark the Earth surface models^2,3^. In particular, it is possible to construct a carbon-water flux simulation model from the station-scale to the regional- or global-scale by means of a large-scale eddy covariance^4^ measurement network (e.g. Fluxnet, AmeriFlux and ChinaFlux). However, the existing flux stations are sparsely and unevenly distributed and yield rather discontinuous observation data^1^. This restricts studies on the carbon-water fluxes at a large-scale^3^, for example in Eurasia, where a strong spatial heterogeneity is exhibited on complex terrains. The meteorological stations, in contrast, are densely spread around the world with long-term continuous observation data^5^, which could have great potential to mine the more extensive carbon-water flux information, particularly combined with machine learning (ML) and remote sensing (RS). This could greatly offset the limitations of the flux station-based observations.

Machine learning is increasingly used to extract the patterns and insights from big geospatial data^6^. Many studies have focused on the comparative evaluation of different ML algorithms and have found the accuracy performance of the same algorithm varies in different research contexts^7–10^. The data-driven ML algorithms are similar to the encapsulated complex empirical algorithms, which demonstrate a high simulation accuracy^3,11^. But the ML algorithms are still influenced by the quality, processing methods, and spatio-temporal representativeness of the data^12–14^. Compared with the process-based land surface or ecosystem models, the ML has a higher carbon-water flux simulation accuracy^1,6^. However, when transferred to other site or regional or spatial (grid) scales, the applicability of both the ML models and process models need to be evaluated due to the distinct spatio-temporal heterogeneity. That is to say, there is no guarantee that these models are applicable to all sites, grids or regions. If this evaluation of the model applicability is not considered, the simulation results will generate new uncertainties. This issue has become a major problem affecting the simulation accuracy of the carbon-water fluxes at different scales.

In this study, ML with flux observations was used to build carbon-water flux simulation models (random forest model, RFM) to simulate the carbon-water fluxes of the meteorological stations in Eurasia. We proposed a framework to evaluate the applicability of the model transfer and to build a bridge from the flux stations to the meteorological stations. We used this framework to generate four carbon-water flux datasets for the Eurasian meteorological stations. Due to the high precision, these datasets could be regarded as quasi-observational at the site level, which might be used to assess the simulation accuracy of the regional- or global-scale ecosystem carbon-water fluxes based on the ecosystem or land surface or remote sensing or atmospheric inversion models. Our study can, therefore, benefit terrestrial water management and carbon dynamic assessments.

## Methods

**Fig. 1:**
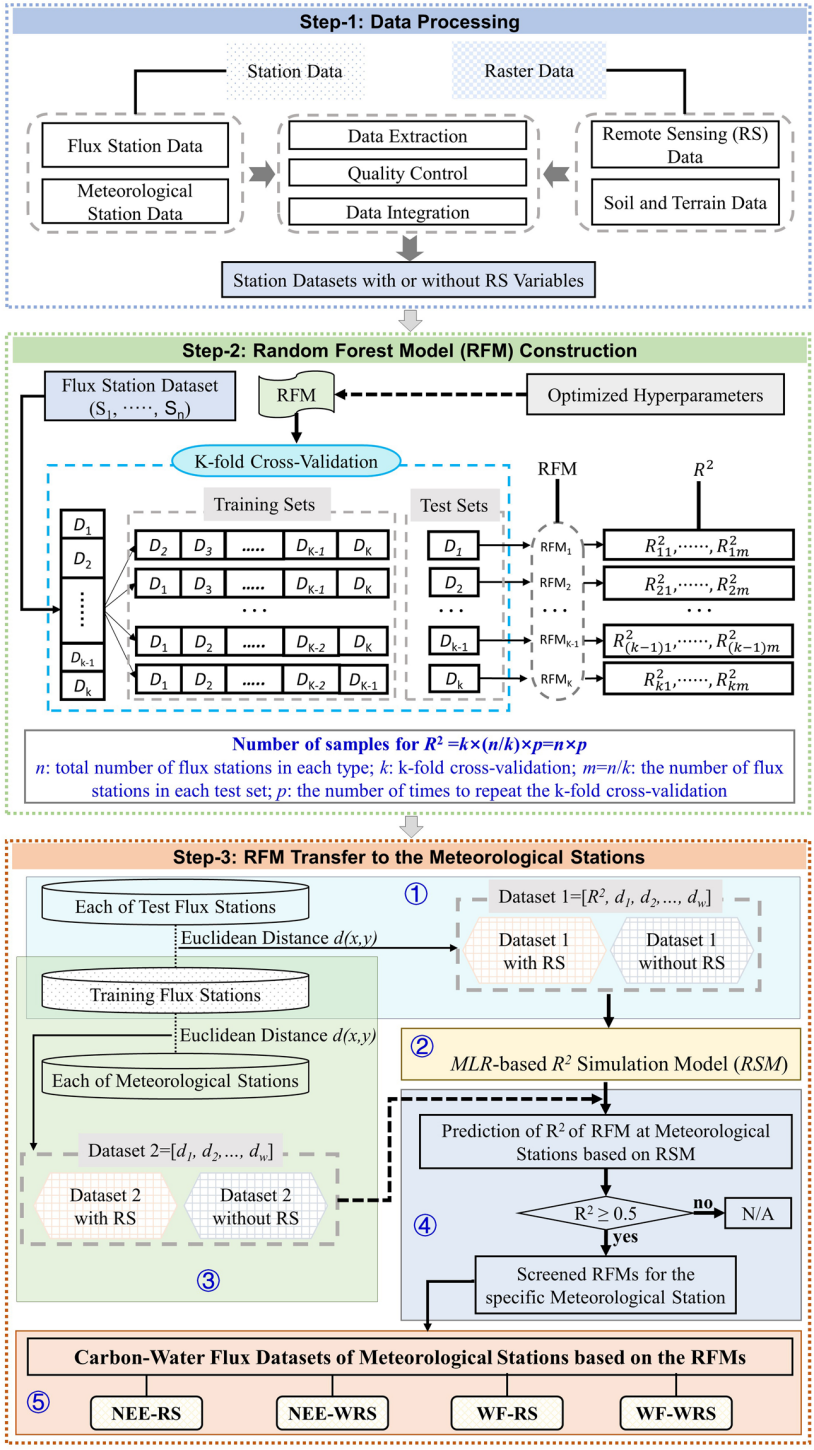
Research framework. R^2^, determination coefficient; MLR, multiple linear regression. N/A (not applicable) indicates that the RFM could not be transferred to the specific meteorological stations. NEE-RS: net ecosystem carbon dioxide exchange (NEE) based on the RFM with remote sensing (RS); WF-RS: water flux (WF) based on the RFM with RS; these explain the fact that the RS variables were used in the RFM construction. NEE-WRS: NEE based on the RFM without RS; WF-WRS: WF based on the RFM without RS; these demonstrate that the RS variables were not applied in the RFM construction. RS variables include the fraction of the photosynthetically active radiation, enhanced vegetation index, land surface water index and surface reflectance for the Moderate Resolution Imaging Spectroradiometer bands 1–7.

The RFM was constructed based on the Eurasian flux stations. We built a total of 3,600 RFMs at site scale in accordance with the classification of the flux stations. The simulation accuracy of these RFMs at each flux station in the test set was validated by the spatial cross-validation, thus generating thousands of determination coefficients (R^2^) at test stations. According to the third law of geography^15^, the factors (variables) similarity between the test station and the training set of the RFM determines the similarity between their fluxes, that is, the R^2^ of the RFM at the test station are determined. The similarity between the datasets composed of the same factors could be characterized by the Euclidean distance. Based on the R^2^ and Euclidean distance, the R^2^ simulation model (RSM) was built by using multiple linear regression (MLR) to evaluate the applicability of RFM on meteorological stations. So that the RFMs can be reasonably transferred to meteorological stations to simulate the carbon-water fluxes. Figure 1 shows the detailed flowchart of the data processing, RFM construction and RFM transfer to the meteorological stations.

### Data processing

**Fig. 2:**
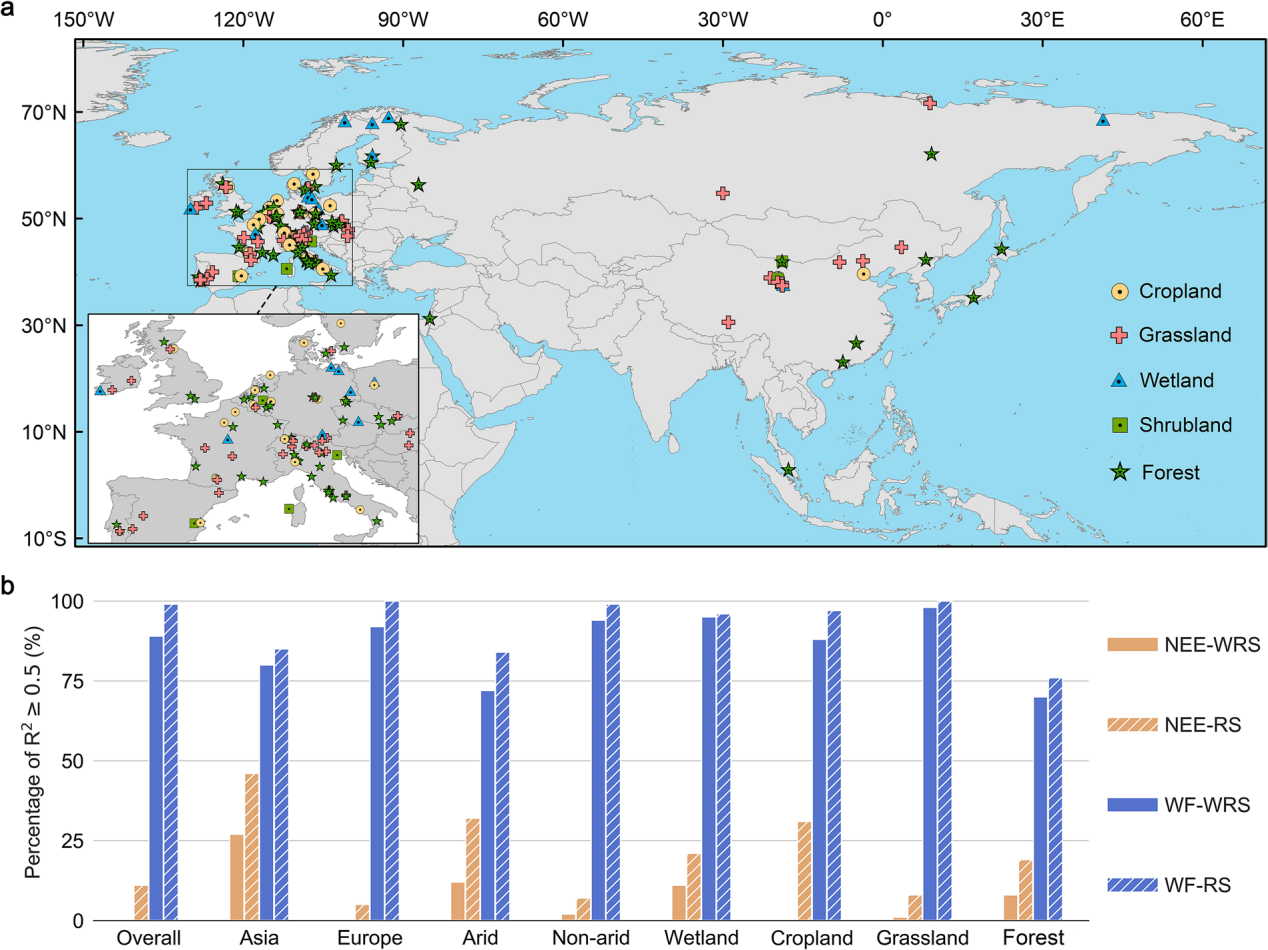
Study area and the accuracy of carbon-water flux simulation models (random forest model, RFM). (**a**) Distribution of the 156 Eurasian flux stations covering five main landscape types. (**b**) The accuracy assessments of the RFM based on the Eurasian flux stations in the framework of the 10-time 10-fold cross-validation. The figure shows the percentage of the RFMs with R^2^ ≥ 0.5 tested in the test sets for nine categories.

We selected 156 flux stations in Eurasia from five different landscape types (Fig. 2a), as detailed in the flux station information file^16^. For the flux stations from the National Tibetan Plateau Data Center (NTPDC)^17–133^ and European Fluxes Database Cluster (EFDC)^134–137^ (http://www.europe-fluxdata.eu/home), the flux data from one hour before (and after) rainfall were excluded. The data collected at 10-min or 30-min intervals were interpolated using the marginal distribution sampling (MDS) method in REddyProc^138^. All final data were converted into daily data. For the flux stations from FLUXNET^139–162^, the data were extracted with quality control values ≥ 0.8 for the net ecosystem carbon dioxide exchange (NEE) and latent heat fluxes (LE). The water fluxes (WF) were converted from LE (W/m^2^) with a conversion factor of 0.408 × 10^−6 ^^163–165^. For the selected 6856 meteorological stations from the Global Surface Summary of the Day dataset in the National Centers for Environmental Information (https://www.ncei.noaa.gov/metadata/geoportal/rest/metadata/item/gov.noaa.ncdc%3AC00516/html#), the vapour pressure deficit variable was calculated using the air temperature and dew point temperature. The downward shortwave radiation (DSR) of meteorological stations for 2002–2020 and 1983–2018 were extracted from the GLASS dataset^166,167^ and the dataset of high-resolution global surface solar radiation^168,169^ from the NTPDC, respectively. For the remote sensing (RS) variables (including the fraction of the photosynthetically active radiation extracted from the MCD15A3H data^170^, enhanced vegetation index, land surface water index and surface reflectance for the Moderate Resolution Imaging Spectroradiometer bands 1–7 extracted from the MOD09GA data^171^), a linear interpolation was carried out for the missing data with continuous missing days < 8^165,172^. Terrain and soil variables were extracted from the MERIT DEM data^173^ and the HWSD data^174^, respectively. High quality RS variables, terrain variables and soil variables averaged over a 500-meter spatial extent centered on the station were integrated into the flux stations and meteorological stations (Table S1).

Due to the significant spatial heterogeneity of the earth’s surface, the flux stations and meteorological stations were divided into nine categories according to the following four strategies. The first is based on the International Geosphere-Biosphere Programme classification from the MCD12Q1 data^175^, including Wetland (i.e. permanent wetlands), Cropland (i.e. croplands and cropland/natural vegetation mosaics), Grassland (i.e. grasslands, savannas, woody savannas) and Forest (i.e. evergreen needleleaf forests, evergreen broadleaf forests, deciduous needleleaf forests, deciduous broadleaf forests, and mixed forests). The second is based on the continents, e.g. Asia and Europe. The third is the arid and non-arid regions classified by the dryland dataset^176^. This dataset identified areas with an aridity index (AI) less than 0.65 as drylands, which were described as arid in this study, and the remaining areas (i.e. AI ≥ 0.65) were classified as non-arid regions. The fourth comprises entire Eurasia, that is, overall. We used the datasets of nine categories from flux stations as input to RFM, and the detail is presented in the division of flux stations file^177^.

### RFM construction

In this work, the random forest method^6,178^ was used to construct the RFM using the scikit-learn library in Python 3.7.6 (https://pypi.org/project/scikit-learn/1.0.1/). The RFM is a combination model based on independent regression trees^179^, of which the predictions were made by averaging the results across all regression trees. The random-search optimizer^180^ was applied to identify the optimal hyperparameter settings for the RFM (Table S2). In addition, the most important step in the RFM construction is the k-fold cross-validation (CV)^181^. Suppose the flux station dataset is composed of data from *n* flux stations and it might generate *k* sub datasets (*D*_1_, *D*_*2*_, …, *D*_*k*_) if it is equally divided into *k* parts. Here, each sub dataset is a test set, which is composed of data from the *m* = *n/k* flux stations, while the remaining sub datasets constitute training sets, which are composed of data from the (*n*-*m*) flux stations. Thus, each training set could be used to establish the RFM for the flux simulation; consequently, a total of *k* models (*RFM*_1_, *RFM*_*2*_, …, *RFM*_*k*_) were built. Each RFM is tested and verified at each flux station in the test set. Furthermore, the R^2^ is calculated for each case. Hence, the R^2^ amount that could be generated after each RFM training and test is given by *k* × *m* = *k* × *n*/*k* = *n*. The flux station dataset (*n*) requires multiple (*p*) repetitions of the k-fold division to avoid the contingency of the station division. In this way, *k* × *p* RFMs could be constructed and the number of R^2^ that could be generated is *n* × *p*.

According to the above-mentioned principles, we used the 10-time (split) 10-fold CV (i.e. *p* = 10 and *k* = 10) to set up 100 RFMs for each of the nine categories under two scenarios: with and without RS variables in the model building (i.e. RFM-RS and RFM-WRS), respectively. That is, we constructed 900 RFM-RS models to simulate NEE and WF, respectively. And we also constructed 900 RFM-WRS models to simulate NEE and WF, respectively. Each RFM was validated at each flux station in the corresponding test set, and the R^2^ was generated to represent the validation accuracy of the RFM. The R^2^ also represents the applicability of these models in the test flux stations. The higher the R^2^ of the model on the test flux station, the more suitable the model is for the specific flux station, that is, the more similar the data characteristics of the training set for building the model are to the data characteristics of the flux station according to the third law of geography.

### RFM transfer to the meteorological stations

We screened available RFMs for meteorological stations by using the RSM, which was used to evaluate the RFM applicability on the meteorological stations. The framework for the evaluation was designed (Fig. 1) as follows:

① *Euclidean distances of the influencing factors between test flux stations and training sets of RFMs*.

The R^2^ of each RFM is determined by the similarity of the influencing factors between the training set and the test set^181^. This could be characterized by the Euclidean distance^182^.

For a specific factor affecting the RFM, the Euclidean distance (*ds*) between a flux station in the test set (test station) and a flux station in the training set (training station) is expressed as:

1 $$ds(x,y)=\sqrt{{({x}_{1}-{y}_{1})}^{2}+{({x}_{2}-{y}_{2})}^{2}+\cdots +{({x}_{t}-{y}_{t})}^{2}}=\sqrt{{\sum }_{(i=1)}^{t}{({x}_{i}-{y}_{i})}^{2}},t\le 365$$

where *x* represents one of the factors influencing the carbon-water fluxes in the training station (Table S1), *y* denotes the corresponding influencing factor in the test station, and *t* is the sample size of the factor. The data of the training station and test station were averaged on the same day (day of the year, DOY) for multiple years, respectively. Then, the two stations could be matched day by day based on DOY to ensure that these have the same daily data sample size.

For the factor *j* influencing the RFM, the final Euclidean distance (*d*) is the average of all *n*-*m ds* between the test station and each of the n-m training stations, which is the Euclidean distance (*d*_*j*_) of the factor *j* between this test station and the training set of the RFM (Eq. 2). In the same way, the Euclidean distances of all influencing factors are produced and denoted as *d*_*1*_, …, *d*_*w-1*_, *d*_*w*_. In this way, the *R*^2^ of the RFM tested in the test station from the test set and the Euclidean distances *d*_*1*_, …, *d*_*w-1*_, *d*_*w*_ between this test station and the training set for building the RFM constitute a complete data sample (Fig. S3). Similarly, all test stations could generate such samples, which constitute a dataset with a quantity equal to 10 × *n* × 9. Samples of the same category are integrated into one dataset, that is, nine datasets produced under nine categories, i.e. Dataset 1 in Fig. 1.

2 $$d(x,y)=\frac{d{s}_{1}(x,y)+d{s}_{2}(x,y)+\cdots +d{s}_{(n-m)}(x,y)}{(n-m)}=\frac{{\sum }_{(i=1)}^{(n-m)}d{s}_{i}(x,y)}{(n-m)}$$

where *n* represents the number of the flux stations of each category and *m* illustrates the number of the flux stations in each test set.

② *Construction of the RSM*.

Based on Dataset 1, the RSM is constructed using MLR^183^ for nine categories under NEE and WF scenarios, of which each one is expressed as:

3 $${R}^{2}={a}_{0}+{a}_{1}{d}_{1}+{a}_{2}{d}_{2}+\cdots +{a}_{(w-1)}{d}_{(w-1)}+{a}_{w}{d}_{w}$$

where *a*_0_, *a*_1_, *a*_2_, …, *a*_*w-1*_, *a*_*w*_ are regression coefficients and *d*_1_, *d*_2_, …, *d*_*w*-1_, *d*_*w*_ indicate the Euclidean distances of the factors influencing the carbon-water fluxes between the test station and the training set.

③ *Euclidean distances of the influencing factors between meteorological stations and training sets of RFMs*.

The same processes of ① are applied to the meteorological stations so as to calculate the Euclidean distance for the influencing factors between each RFM training set and meteorological station for each category under two scenarios, which yields a large dataset, i.e. Dataset 2 in Fig. 1.

④ *Prediction of the R*^2^ *of the RFM transfer to the meteorological stations*.

Before a RFM is transferred to a specific meteorological station, the RSM could predict the R^2^ value on the station using Dataset 2 in Fig. 1. Only if predicted R^2^ ≥ 0.5, its corresponding RFM might be transferred to the corresponding meteorological stations. Otherwise, the RFM was assumed to be not applicable to the meteorological station. If there was more than one RFM applicable to a meteorological station, the RFM corresponding to the maximum predicted R^2^ was screened as the model that could be linked to the meteorological station. Not all meteorological stations did have a RFM which is applicable to the meteorological station.

⑤ *Carbon-water flux simulation of the meteorological stations*.

For the meteorological stations in Eurasia that could be linked with an applicable RFM, the corresponding RFM can be used to simulate the daily carbon-water fluxes and to build high-precision carbon-water flux datasets of the Eurasian meteorological stations to analyze the carbon-water dynamics. These datasets^184^ consist of two essential building blocks: (i) datasets related to remote sensing, including the net ecosystem carbon dioxide exchange (NEE-RS) and water fluxes (WF-RS) simulated by the RFM-RS; (ii) the net ecosystem carbon dioxide exchange (NEE-WRS) and water fluxes (WF-WRS) simulated by the RFM-WRS.

## Data Records

Our carbon-water flux datasets^184^ are available at figshare (10.6084/m9.figshare.21347721.v3). The data record contains two daily carbon dioxide flux datasets (NEE-RS and NEE-WRS) and two daily water flux datasets (WF-RS and WF-WRS) of the Eurasian meteorological stations. The coverage period of the NEE-RS and WF-RS has been recorded from 2002 to 2020 and the one of NEE-WRS and WF-WRS from 1983 to 2018. The data of each meteorological station was deposited separately in the CSV file format under the dataset folders. The file name indicates the identification number of the meteorological station in the meteorological station information file^185^ (10.6084/m9.figshare.23695920.v2). The list of flux stations used in this study and the details of flux station division used for the RFM construction are shown in the flux station information file^16^ (10.6084/m9.figshare.23899701.v1) and the division of flux stations file^177^ (10.6084/m9.figshare.23695980.v2) stored at figshare, respectively. In addition, the details of the RSM construction are presented in the RSMs information file^186^ (10.6084/m9.figshare.23899785.v1) deposited at figshare. The file specific fields are as follows:

### Carbon-water flux datasets file (.csv)

1. id: Identification of the meteorological station.
2. lon: Longitude of the meteorological station.
3. lat: Latitude of the meteorological station.
4. year: Year of the data record.
5. month: Month of the data record.
6. day: Day of the data record.
7. doy: Day of the year.
8. NEE: Net ecosystem carbon dioxide exchange (g C m^−2^ d^−1^).
9. WF: Water fluxes (mm d^−1^).

### Meteorological station information file (.xlsx)

1. Identification of meteorological station.
2. Station name: Name of the meteorological station.
3. Longitude: Longitude of the meteorological station.
4. Latitude: Latitude of the meteorological station.
5. Elevation: Elevation of the meteorological station (m).
6. Continent: Continent of the meteorological station.
7. Drought situation: Drought situation of the meteorological station.
8. Landscape: Landscape of the meteorological station.
9. Data source: Data source of the meteorological station.
10. Classification of simulated NEE-RS: Accuracy classification of NEE-RS for meteorological stations (1: low quality, R^2^ < 0.5; 2: moderate quality, 0.5 ≤ R^2^ < 0.7; 3: high quality, R^2^ ≥ 0.7).
11. Classification of simulated NEE-WRS: Accuracy classification of NEE-WRS for meteorological stations (1: low quality, R^2^ < 0.5; 2: moderate quality, 0.5 ≤ R^2^ < 0.7; 3: high quality, R^2^ ≥ 0.7).
12. Classification of simulated WF-RS: Accuracy classification of WF-RS for meteorological stations (1: low quality, R^2^ < 0.5; 2: moderate quality, 0.5 ≤ R^2^ < 0.7; 3: high quality, R^2^ ≥ 0.7).
13. Classification of simulated WF-WRS: Accuracy classification of WF-WRS for meteorological stations (1: low quality, R^2^ < 0.5; 2: moderate quality, 0.5 ≤ R^2^ < 0.7; 3: high quality, R^2^ ≥ 0.7).

### Flux station information file (.xlsx)

1. Identification of flux stations.
2. Flux station: Name of the flux station.
3. Longitude: Longitude of the flux station.
4. Latitude: Latitude of the flux station.
5. Elevation: Elevation of the flux station (m).
6. Continent: Continent of the flux station.
7. Drought situation: Drought situation of the flux station.
8. Landscape: Landscape of the flux station.
9. Study period: Study period of the flux station used in this study.
10. Data source: Data source of the flux station.

### Division of flux stations file (.xlsx)

1. Categories: Category of the flux station.
2. Number of flux station: Number of flux stations under each category.
3. Split: Identification of divisions for 10-fold cross-validation on flux stations.
4. Fold: Identification of folds for cross-validation on flux stations.
5. Identification of flux station: List of identifications for flux stations under each fold.

### RSMs information file (.xlsx)

1. Models: Name of the RSM.
2. Categories: Category of the RSM.
3. N: Number of samples used by the RSM.
4. R^2^_rsm_: Determination coefficient of the RSM.
5. Adj. R^2^_rsm_: Adjusted determination coefficient of the RSM.
6. F-statistic: F-statistic of the RSM.
7. P value: Significance probability of the RSM.
8. RSMs: Equation of the RSM.

## Technical Validation

### Model validation

The R^2^ and RMSE (root mean square error) were used to evaluate the performance of the RFM to simulate the NEE-RS (NEE simulated by RFM-RS), WF-RS (WF simulated by RFM-RS), NEE-WRS (NEE simulated by RFM-WRS) and WF-WRS (WF simulated by RFM-WRS)^184^. The model’s simulation accuracy for the WF was much higher than for NEE under each category and the performance of the RFM-RS was also better than the RFM-WRS (Fig. 2b, Table S3). For the WF simulation of the RFM-RS and RFM-WRS under each category, the percentage of the models with R^2^ ≥ 0.5 in the test sets was larger than 70%, while for the NEE simulation, it was lower than 50% (Fig. 2b). For the category ‘overall’, the RFM generally indicated a high simulation accuracy (Fig. 2b, Table S3). The simulation accuracy of the RFM was generally higher in Asia and the arid regions than in Europe and the non-arid regions. For the cropland and forest, the RFMs demonstrated the highest simulation accuracy under the scenarios of NEE-RS and NEE-WRS; for the grassland and wetland, the RFMs demonstrated the highest simulation accuracy under the WF-RS and WF-WRS scenarios.

**Fig. 3:**
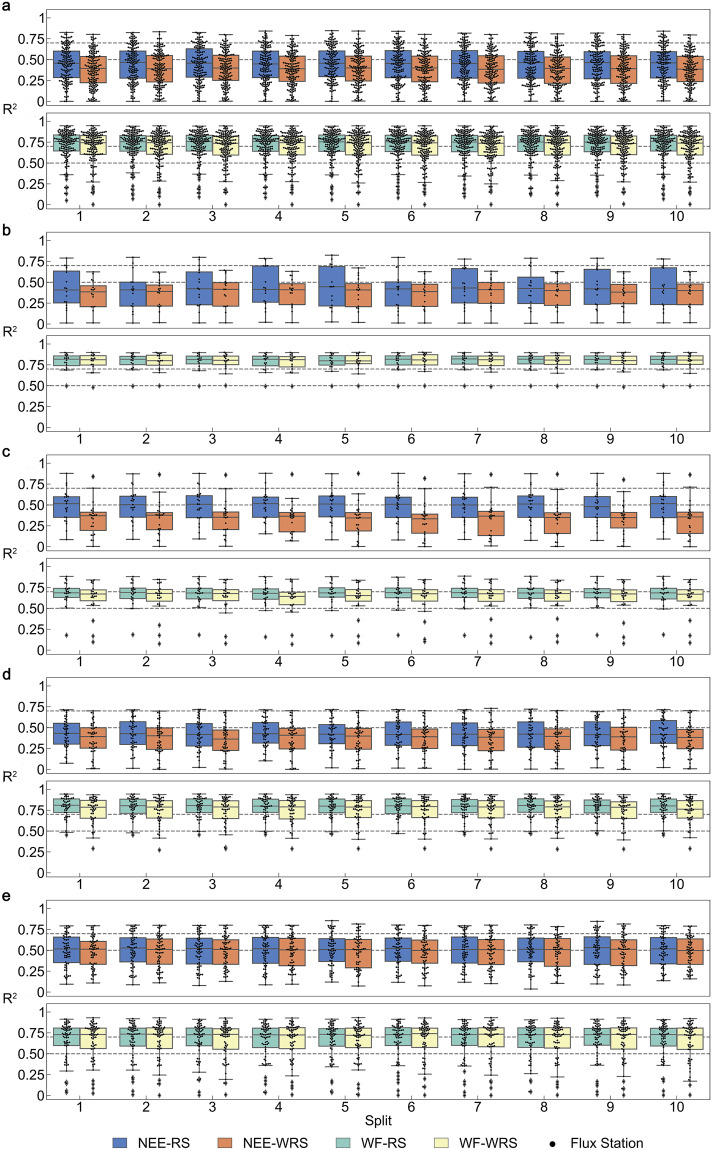
The accuracy performance of the carbon-water flux simulation models (random forest model, RFM) at test flux stations. The NEE (net ecosystem carbon dioxide exchange) and WF (water flux) R^2^-based accuracy performance of the RFM of each split of the 10-time 10-fold cross-validation for (**a**) Overall with 156 stations, (**b**) Wetland with 16 stations, (**c**) Cropland with 23 stations, (**d**) Grassland with 47 stations and (**e**) Forest with 64 stations. The box plots show the R^2^ distribution of each flux station of the test set for different categories, in which the whiskers indicate the 1.5 times’ interquartile range.

The box plots (Fig. 3, Fig. S1) present the simulation performance of the RFM for NEE and WF in 10-time 10-fold CVs, with each box representing the R^2^ distribution for the test flux stations in each split (time). The simulation accuracy of the same RFM for different test flux stations varied widely, indicating that the RFM cannot be applied to all flux stations and that not all stations could match at least one available RFM model. The maximum R^2^ distribution for each flux station was observed in a 10-time 10-fold CV (Table S4). The proportion of the flux stations with R^2^ ≥ 0.5 of the RFM test measured 60.9%, 46.2%, 89.7% and 88.5% under the NEE-RS, NEE-WRS, WF-RS and WF-WRS scenarios, respectively.

### Accuracy pre-assessment of the carbon-water flux simulation at the meteorological stations

Using the MLR model in which the Euclidean distances were independent variables and R^2^ concerned a dependent variable, the RSMs were constructed for different categories under different scenarios, as shown in the RFMs information file^186^. By using the RSM to simulate the R^2^ of the RFM at the test flux stations, the overall accuracies (of the RSM) for a correct classification of R^2^ under nine categories amounted to 80.1%, 84.0%, 91.0% and 89.1% for the NEE-RS, NEE-WRS, WF-RS and WF-WRS, respectively (Fig. S2). This might prove that the RSM is reliable and could be utilized to predict the accuracy of the RFMs applied to the meteorological stations.

**Fig. 4:**
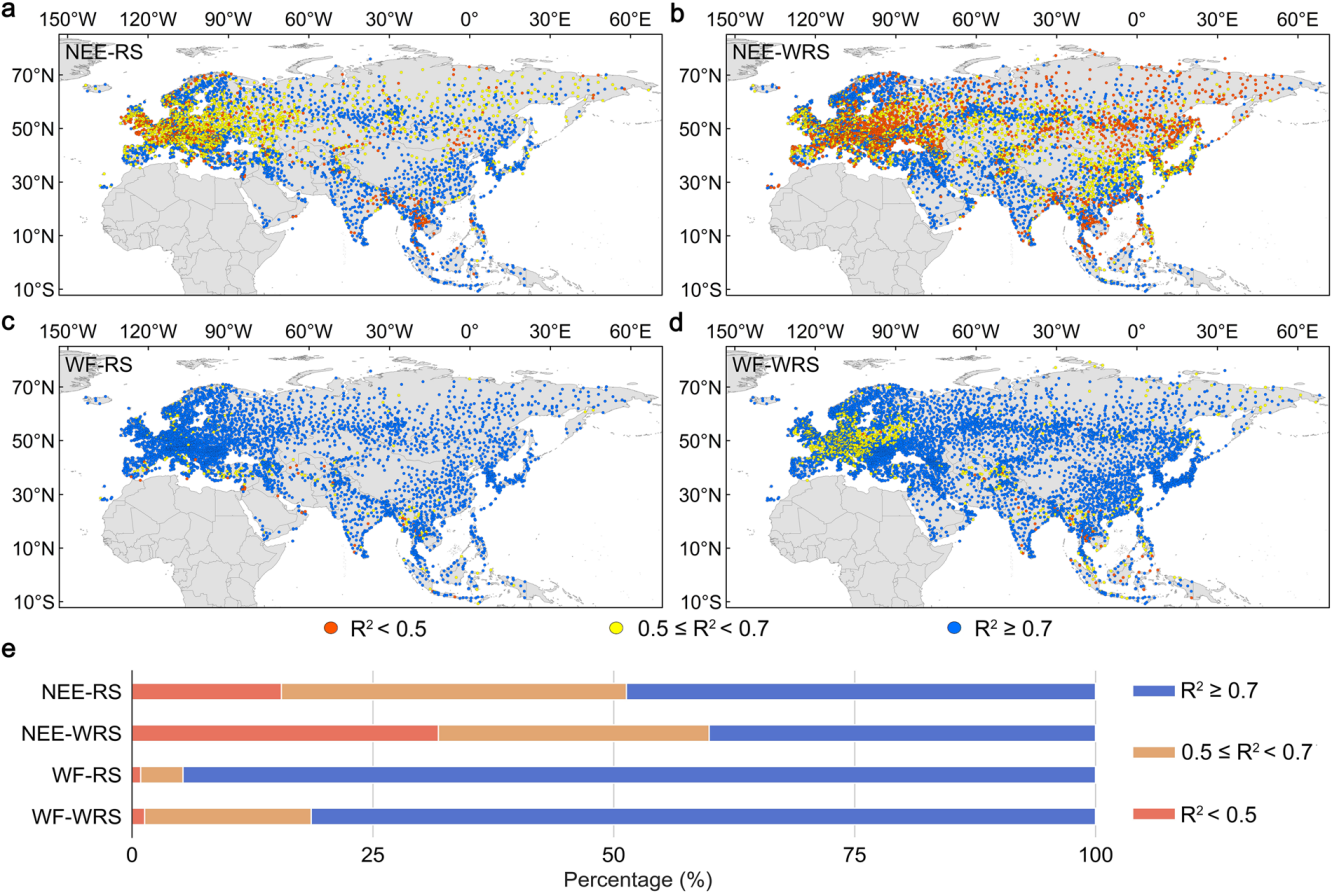
The distribution of the R^2^ predicted by the R^2^ simulation model (RSM) at the meteorological stations. Spatial distribution of the R^2^ at (**a**) 4466 meteorological stations under the scenario of NEE-RS, (**b**) 6849 meteorological stations under the scenario of NEE-WRS, (**c**) 4466 meteorological stations under the scenario of WF-RS and (**d**) 6849 meteorological stations under the scenario of WF-WRS, respectively. **(e**) The percentage distribution of R^2^ < 0.5, 0.5 ≤ R^2^ < 0.7 and R^2^ ≥ 0.7 of the meteorological stations in the different scenarios.

Finally, the RFMs were transferred to all meteorological stations in Eurasia and the R^2^ was predicted for every meteorological station for each category under two scenarios (Fig. 4a–d, Table S5). In this study, the criteria for screening the RFM imply that the RFM corresponding to the highest predicted R^2^ of a given meteorological station and its R^2^ ≥ 0.5 was screened as the simulation model of the carbon-water fluxes for the meteorological station. The percentages of the meteorological stations in Eurasia were 84.5%, 68.2%, 99.1% and 98.7% for the NEE-RS, NEE-WRS, WF-RS and WF-WRS, respectively, in which the RFMs met the above-mentioned criteria (Fig. 4e). The RFMs have much higher applicable percentages and seem more accurate for the WF simulation than the NEE simulation at the meteorological stations. The RFM models of the forest and grassland categories were highly applicable and more accurate regarding the NEE and WF simulation of the meteorological stations than for cropland or wetland (Table S5).

The input data for the RFM were primarily derived from the meteorological stations’ observations and remote sensing data. Moreover, machine learning models (such as RFM) have the advantage and the predictive ability in the non-linear relation fitting and have been proven in the application research of relevant geoscience^6^, which is generally superior to linear regression, ecosystem process models, remote sensing inversion models, etc. Therefore, the carbon-water flux datasets of the meteorological stations generated in this study demonstrate a relatively high accuracy and constitute the attribute of quasi-observation, which might be considered as a quasi-observational dataset. They could be applied as benchmark data to verify the simulation results produced by the process-based models or remote sensing inversion models related to the carbon-water fluxes, which overcomes the challenge of insufficient observational data on the carbon-water fluxes^165,187^.

### Spatio-temporal patterns of the Eurasian NEE and WF

**Fig. 5:**
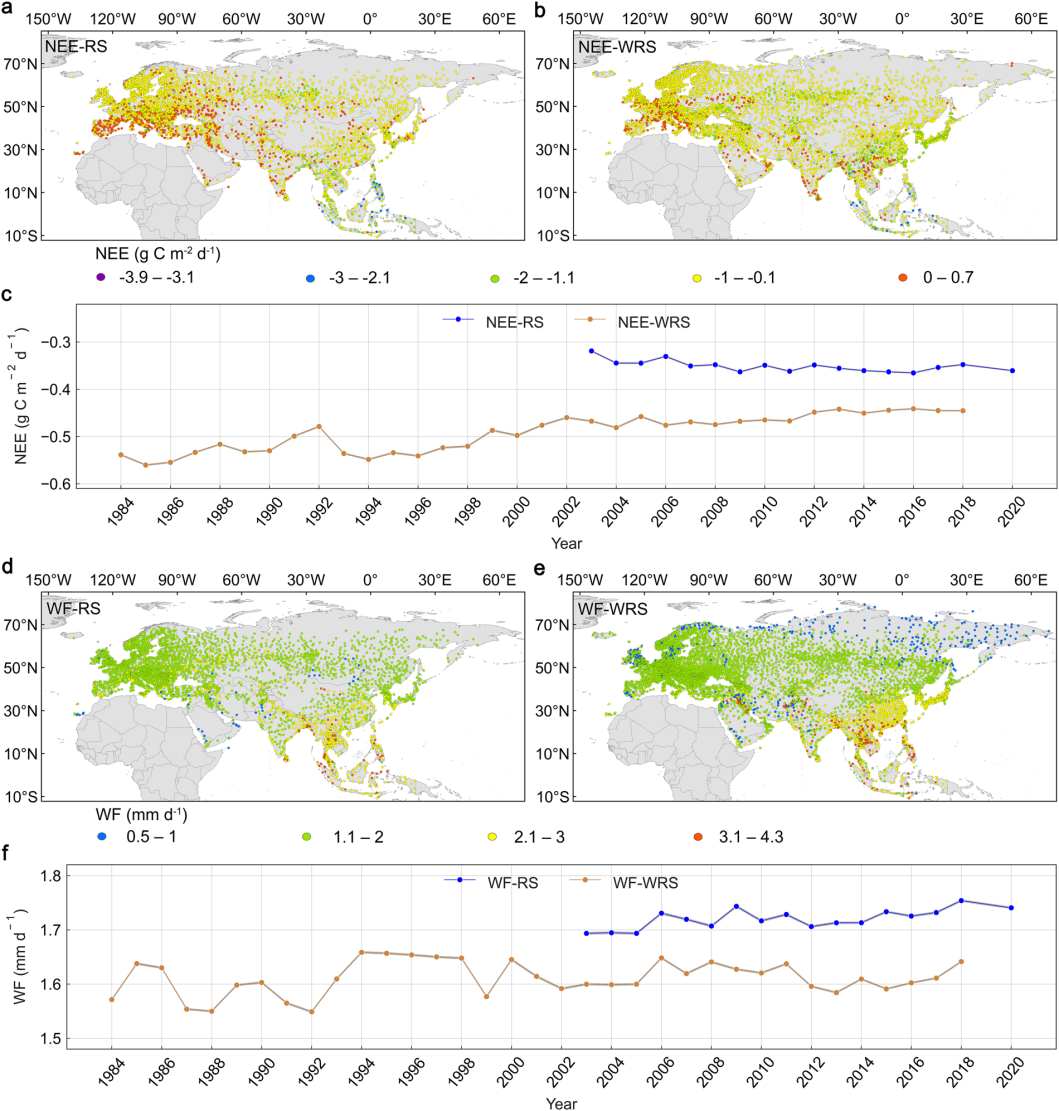
Spatio-temporal variations of the carbon-water fluxes at the Eurasian meteorological stations. Spatial distribution of the mean daily values during the period March-November of (**a**) NEE-RS from 2003 to 2020 for 3436 meteorological stations and (**b**) the NEE-WRS from 1984 to 2018 for 4352 stations. (**c)** The annual temporal variation of the mean daily NEE (net ecosystem carbon dioxide exchange) values for the meteorological stations and the corresponding 95% confidence interval shown as a shaded band. Spatial distribution of the mean daily values during the period March-November of (**d**) WF-RS from 2003 to 2020 for 3990 stations and (**e**) WF-WRS from 1984 to 2018 for 6302 stations. (**f**) The annual temporal variation of the mean daily WF (water flux) values for the meteorological stations and the corresponding 95% confidence interval shown as a shaded band.

We have further investigated the spatio-temporal distribution of the mean daily values of NEE and WF simulated by the RFM-RS during the period March-November from 2003 to 2020 and simulated by the RFM-WRS from 1984 to 2018. The meteorological stations with at least 30 data volumes of each spring, summer and autumn were used for statistical analysis. The mean daily values of the NEE-RS, NEE-WRS, WF-RS and WF-WRS at the meteorological stations are −3.9~0.7 g C m^−2^ d^−1^, −2.6~0.4 g C m^−2^ d^−1^, 0.8~3.8 mm d^−1^ and 0.5~4.3 mm d^−1^, respectively (Fig. 5a,b,d,e). The spatial distribution of these mean daily NEE fluxes reveals that the ecosystem carbon dioxide loss had increased in Eurasia during 2003–2020, with 457 carbon dioxide loss stations during this period, which means 178 more than from 1984 to 2018 (Fig. 5a,b). The daily average NEE (generally presented as net carbon dioxide uptake) has shown an increasing trend from 1984 to 2002, while a slightly decreasing tendency from 2003 to 2020, with slight fluctuations during these two periods (Fig. 5c). The temporal variation of the WF has demonstrated a rising trend with a distinct fluctuation from 1984 to 2020 (Fig. 5f). The differences between RS and WRS products might be caused by the differences in the input DSR dataset, the RFMs, and the number of meteorological stations (Fig. 5c,f).

### Comparison of the NEE and WF with other carbon-water flux products

**Fig. 6:**
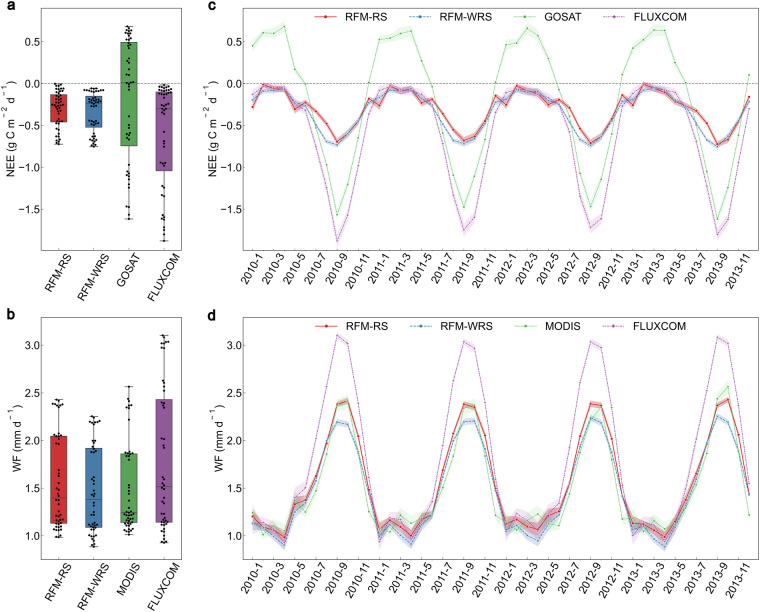
Comparison of the monthly NEE (net ecosystem carbon dioxide exchange) and WF (water flux) in this study with those from GOSAT, MODIS and FLUXCOM during the period 2010–2013. The box plots of the monthly values (black dots) for (**a**) NEE and (**b**) WF, respectively, in which the whiskers indicate the 1.5 times’ interquartile range. The monthly changes in (**c**) NEE and (**d**) WF and the corresponding 95% confidence interval shown as a coloured line and shaded band, respectively. RFM-RS: NEE or WF based on the RFM with remote sensing (RS), representing the fact that the RS variables were used in the RFM construction. RFM-WRS: NEE or WF based on the RFM without RS, illustrating that the RS variables were not used in the RFM construction. The RS variables include a fraction of the photosynthetically active radiation, enhanced vegetation index, land surface water index and surface reflectance for the Moderate Resolution Imaging Spectroradiometer bands 1–7. GOSAT, the GOSAT L4A data; MODIS, the MOD16A2 Version 6 data; FLUXCOM, an initiative to upscale the biosphere-atmosphere fluxes from the FLUXNET sites to the continental and global scales.

We further compared the NEE and WF datasets with those from FLUXCOM^1,3^, GOSAT L4A (https://data2.gosat.nies.go.jp/) and MODIS (MOD16A2 Version 6)^188^ (Fig. 6, Table S6). The WF from the other products were converted from the LE. Our NEE and WF datasets, as well as the fluxes from the other products, were converted into a monthly scale. The months with the same number of stations for each product were selected for a comparison. All data show a similar seasonal variation, with high carbon-water fluxes in summer and low during winter (Fig. 6c,d). The distributions of the carbon-water fluxes from FLUXCOM and NEE from GOSAT are relatively discrete (Fig. 6a,b). The multi-year monthly averages of the NEE (NEE-RS = −0.31 g C m^−2^ d^−1^ and NEE-WRS = −0.34 g C m^−2^ d^−1^) and WF (WF-RS= +1.57 mm d^−1^ and WF-WRS= +1.48 mm d^−1^) simulated herein are less than those from FLUXCOM (NEE = −0.61 g C m^−2^ d^−1^, and WF= +1.79 mm d^−1^), whereas the averages are larger than those from GOSAT (NEE = −0.20 g C m^−2^ d^−1^) and MODIS (WF= +1.51 mm d^−1^). The WF from MODIS were almost consistent with our results (Fig. 6d). Because of the fact that the carbon-water flux datasets of the meteorological stations (generated by the RFM in this study) could be considered as “quasi-observational data”, the Eurasian carbon-water fluxes from FLUXCOM may be overestimated, while the NEE from GOSAT could rather be underestimated (Fig. 6c,d).

### Supplementary information

Supplementary Information

## Data Availability

The code^189^ to generate the carbon-water flux datasets is available at figshare (10.6084/m9.figshare.21510183.v2).
