# Supplementary material for: Monitoring of carbon-water fluxes at Eurasian meteorological stations using random forest and remote sensing
Source: Sci Data. 2023 Sep 7;10:587. doi: 10.1038/s41597-023-02473-9 (PMC10485062; doi:10.1038/s41597-023-02473-9)
Supplement: Supplementary file 1 — Supplementary Information [file 41597_2023_2473_MOESM1_ESM.pdf]

Supplementary Information for "Monitoring of carbon-water fluxes at Eurasian meteorological stations using random forest and remote sensing"

Mingjuan Xie, Xiaofei Ma, Yuangang Wang, Chaofan Li, Haiyang Shi, Xiuliang Yuan, Olaf Hellwich, Chunbo Chen, Wenqiang Zhang, Chen Zhang, Qing Ling, Ruixiang Gao, Yu Zhang, Friday Uchenna Ochege, Amaury Frankl, Philippe De Maeyer, Nina Buchmann, Iris Feigenwinter, Jørgen E. Olesen, Radoslaw Juszczak, Adrien Jacotot, Aino Korrensalo, Andrea Pitacco, Andrej Varlagin, Ankit Shekhar, Annalea Lohila, Arnaud Carrara, Aurore Brut, Bart Kruijt, Benjamin Loubet, Bernard Heinesch, Bogdan Chojnicki, Carole Helfter, Caroline Vincke, Changliang Shao, Christian Bernhofer, Christian Brümmer, Christian Wille, Eeva-Stiina Tuittila, Eiko Nemitz, Franco Meggio, Gang Dong, Gary Lanigan, Georg Niedrist, Georg Wohlfahrt, Guoyi Zhou, Ignacio Goded, Thomas Gruenwald, Janusz Olejnik, Joachim Jansen, Johan Neirynck, Juha-Pekka Tuovinen, Junhui Zhang, Katja KLUMPP, Kim Pilegaard, Ladislav Šigut, Leif Klemedtsson, Luca Tezza, Lukas Hörtnagl, Marek Urbaniak, Marilyn Roland, Marius Schmidt, Mark A. Sutton, Markus Hehn, Matthew Saunders, Matthias Mauder, Mika Aurela, Mika Korkiakoski, Mingyuan Du, Nadia Vendrame, Natalia Kowalska, Paul G. Leahy, Pavel Alekseychik, Peili Shi, Per Weslien, Shiping Chen, Silvano Fares, Thomas Friborg, Tiphaine Tallec, Tomomichi Kato, Torsten Sachs, Trofim Maximov, Umberto Morra di Cella, Uta Moderow, Yingnian Li, Yongtao He, Yoshiko Kosugi and Geping Luo

Contents

**Name**	**Page number**
Table S1	1
Table S2	2
Table S3	3
Table S4	4
Table S5	5
Table S6	5
Fig. S1	6
Fig. S2	7
Fig. S3	7

Table S1. Variables affecting carbon-water fluxes.

Variable	Description (units)	SR	TR	Data source
Lon	Longitude (°)	Point	Daily	Extracted from observation datasets for each flux station (http://data.tpdc.ac.cn; http://www.europe-fluxdata.eu/home; https://fluxnet.org) and meteorological station (https://www.ncei.noaa.gov/metadata/geoportal/rest/metadata/item/gov.noaa.ncdc%3AC00516/html#). Data collected at 10-min or 30-min intervals were converted into a daily-scale.
Lat	Latitude (°)	Point	Daily	
DOY	Day of the year (-)	Point	Daily	
Tmax	Daily maximum temperature (°C)	Point	Daily	
Tmin	Daily minimum temperature (°C)	Point	Daily	
Tmean	Daily average temperature (°C)	Point	Daily	
DTR	Diurnal temperature range (°C)	Point	Daily	
Prcp	Precipitation (mm)	Point	Daily	
WS	Wind speed (m/s)	Point	Daily	
VPD	Vapour pressure deficit (hPa)	Point	Daily	
DSR	Downward shortwave radiation (W/m^2)	Point	Daily	
		0.1°	Daily	Extracted from datasets in National Tibetan Plateau Data Center to each meteorological station during 1983-2018[1, 2].
		0.05°	Daily	Extracted from datasets in GLASS to each meteorological station during 2002-2020[3].
FPAR	Fraction of the photosynthetically active radiation (-)	500m	4-Day	Extracted from MCD15A3H Version 6[4]. Data collected at 4-Day intervals were converted into a daily-scale.
EVI	Enhanced vegetation index (-)	500m	Daily	Calculated using MOD09GA version 6[5].
LSWI	Land surface water index (-)	500m	Daily	
SR_1	Surface reflectance for band 1 (-)	500m	Daily	
SR_2	Surface reflectance for band 2 (-)	500m	Daily	
SR_3	Surface reflectance for band 3 (-)	500m	Daily	
SR_4	Surface reflectance for band 4 (-)	500m	Daily	
SR_5	Surface reflectance for band 5 (-)	500m	Daily	
SR_6	Surface reflectance for band 6 (-)	500m	Daily	
SR_7	Surface reflectance for band 7 (-)	500m	Daily	
Elevation	Elevation at each station (m)	90m	-	Calculated using MERIT DEM[6].
Aspect	Aspect at each station (°)	90m	-	
Slope	Slope at each station (°)	90m	-	
Sand	Percentage of sand (%)	800m	-	Extracted using HWSD version 1.2[7].
Silt	Percentage of silt (%)	800m	-	
Clay	Percentage of clay (%)	800m	-	

SR, spatial resolution; TR, temporal resolution; GLASS, the Global Land Surface Satellite Product. EVI=$2.5 * (\rho_{NIR} - \rho_R)/(\rho_{NIR}+6 * \rho_R - 7.5 * \rho_B+1)$, where ρ_{NIR}, ρ_R and ρ_B are the surface reflectance values of near infrared band, red band and blue band, respectively. LSWI= $(\rho_{NIR} - \rho_{SWIR6})/(\rho_{NIR} + \rho_{SWIR6})$, where ρ_{NIR} and ρ_{SWIR6} are the surface reflectance

values of near infrared band and shortwave infrared for band 6 (SWIR6: 1628 − 1652nm), respectively.

Table S2. Hyperparameter settings of random forest models (RFM) for the carbon-water flux simulation.

Models	Categories	Hyperparameters			
		n_estimators (RS | WRS)	max_depth (RS | WRS)	max_features (RS | WRS)	min_samples_leaf (RS | WRS)
RFM-NEE	Overall	188 | 424	29 | 27	4 | 3	6 | 2
	Asia	416 | 180	13 | 22	9 | 10	3 | 3
	Europe	355 | 216	20 | 29	5 | 5	27 | 29
	Arid	188 | 424	29 | 27	4 | 3	6 | 2
	Non-arid	188 | 424	29 | 27	4 | 3	6 | 2
	Wetland	416 | 424	13 | 27	9 | 3	3 | 2
	Cropland	373 | 216	13 | 29	6 | 5	28 | 29
	Grassland	175 | 451	25 | 27	12 | 5	19 | 15
	Forest	156 | 424	25 | 27	7 | 3	20 | 2
RFM-WF	Overall	329 | 349	25 | 21	12 | 10	21 | 8
	Asia	188 | 424	29 | 27	4 | 3	6 | 2
	Europe	416 | 277	13 | 22	9 | 7	3 | 28
	Arid	188 | 180	29 | 22	4 | 10	6 | 3
	Non-arid	371 | 163	17 | 14	9 | 6	19 | 28
	Wetland	413 | 180	18 | 22	18 | 10	6 | 3
	Cropland	416 | 424	13 | 27	9 | 3	3 | 2
	Grassland	416 | 424	13 | 27	9 | 3	3 | 2
	Forest	216 | 413	29 | 18	21 | 11	29 | 18

NEE, net ecosystem carbon dioxide exchange; WF, water flux; n_estimators, the number of decision trees; max_depth, the maximum depth of the tree; max_features, the number of features to consider when looking for the best split; min_samples_leaf, the minimum number of samples required to be at a leaf node. RS (remote sensing), representing that RS variables were used in random forest models (RFM) construction. WRS (without remote sensing), representing that RS variables were not used in RFM construction. RS variables include the fraction of the photosynthetically active radiation, enhanced vegetation index, land surface water index and surface reflectance for the Moderate Resolution Imaging Spectroradiometer bands 1–7.

Table S3. The mean value of performance indicators on test set in 10-time 10-fold CVs to evaluate the efficacy of carbon-water flux simulation models (random forest models, RFM).

Models	Categories	N	R^2 (STD) (RS)	R^2 (STD) (WRS)	RMSE (STD) (RS)	RMSE (STD) (WRS)
RFM-NEE	Overall	200965	0.37 (0.09)	0.28 (0.08)	0.89 (0.29)	0.95 (0.29)
	Asia	28197	0.44 (0.19)	0.36 (0.19)	1.23 (0.42)	1.34 (0.43)
	Europe	172768	0.35 (0.09)	0.24 (0.07)	0.80 (0.29)	0.87 (0.29)
	Arid	30667	0.39 (0.19)	0.27 (0.18)	0.89 (0.50)	0.96 (0.55)
	Non-arid	170298	0.35 (0.10)	0.28 (0.09)	0.90 (0.27)	0.95 (0.27)
	Wetland	12932	0.32 (0.23)	0.27 (0.18)	1.38 (1.08)	1.31 (1.03)
	Cropland	29063	0.45 (0.14)	0.28 (0.11)	1.05 (0.53)	1.17 (0.50)
	Grassland	57688	0.37 (0.09)	0.31 (0.10)	0.64 (0.17)	0.67 (0.19)
	Forest	93999	0.39 (0.12)	0.33 (0.12)	0.91 (0.40)	0.96 (0.40)
RFM-WF	Overall	200965	0.67 (0.07)	0.61 (0.09)	0.74 (0.10)	0.80 (0.12)
	Asia	28197	0.69 (0.20)	0.64 (0.23)	0.89 (0.37)	1.00 (0.42)
	Europe	172768	0.67 (0.07)	0.63 (0.08)	0.70 (0.07)	0.73 (0.08)
	Arid	30667	0.68 (0.17)	0.61 (0.22)	0.83 (0.32)	0.90 (0.35)
	Non-arid	170298	0.67 (0.07)	0.64 (0.08)	0.71 (0.07)	0.75 (0.08)
	Wetland	12932	0.78 (0.11)	0.78 (0.11)	0.71 (0.42)	0.70 (0.42)
	Cropland	29063	0.66 (0.09)	0.61 (0.13)	0.77 (0.12)	0.83 (0.12)
	Grassland	57688	0.79 (0.07)	0.74 (0.09)	0.59 (0.08)	0.66 (0.09)
	Forest	93999	0.58 (0.11)	0.56 (0.14)	0.81 (0.15)	0.83 (0.16)

NEE, net ecosystem carbon dioxide exchange; WF, water flux; R^2, determination coefficient; RMSE, root mean square error; STD, Standard Deviation; N, number of samples.

Table S4. The distribution of maximum R^2 at each flux station in 10-time 10-fold CVs.

Fluxes	Categories	N	Prec. (R^2<0.5) (RS | WRS)	Prec. (0.5 ≤ R^2<0.7) (RS | WRS)	Prec. (R^2≥0.7) (RS | WRS)
NEE	Overall	156	51.9% | 60.9%	30.8% | 32.7%	17.3% | 6.4%
	Asia	30	43.3% | 53.3%	43.4% | 36.7%	13.3% | 10.0%
	Europe	126	59.5% | 64.3%	23.8% | 30.9%	16.7% | 4.8%
	Arid	28	53.6% | 67.9%	32.1% | 25.0%	14.3% | 7.1%
	Non-arid	128	53.1% | 60.9%	30.5% | 33.6%	16.4% | 5.5%
	Wetland	16	56.2% | 68.7%	12.5% | 31.3%	31.3% | 0%
	Cropland	23	43.5% | 82.6%	43.5% | 8.7%	13.0% | 8.7%
	Grassland	47	51.1% | 70.2%	40.4% | 23.4%	8.5% | 6.4%
	Forest	64	42.2% | 43.7%	34.4% | 37.5%	23.4% | 18.8%
	Total	156	39.1% | 53.8%	40.4% | 32.7%	20.5% | 13.5%
WF	Overall	156	10.9% | 13.5%	23.1% | 26.3%	66.0% | 60.2%
	Asia	30	13.4% | 10.0%	23.3% | 30.0%	63.3% | 60.0%
	Europe	126	12.7% | 13.5%	20.6% | 24.6%	66.7% | 61.9%
	Arid	28	25.0% | 25.0%	7.1% | 17.9%	67.9% | 57.1%
	Non-arid	128	8.6% | 10.9%	25.8% | 28.9%	65.6% | 60.2%
	Wetland	16	6.2% | 0%	6.3% | 12.5%	87.5% | 87.5%
	Cropland	23	4.4% | 13.0%	47.8% | 52.2%	47.8% | 34.8%
	Grassland	47	4.3% | 6.4%	19.1% | 25.5%	76.6% | 68.1%
	Forest	64	20.3% | 20.3%	17.1% | 15.6%	62.5% | 64.1%
	Total	156	10.3% | 11.5%	20.5% | 21.8%	69.2% | 66.7%

Prec., percentage of flux station; N, number of flux stations; R^2, determination coefficient; Total, the maximum R^2 for each flux station was selected for counting under 9 categories.

Table S5. The distribution of maximum R^2 predicted by R^2 simulation model (RSM) at the meteorological stations.

Fluxes	Categories	N (RS | WRS)	Prec. (R^2<0.5) (RS | WRS)	Prec. (0.5 ≤ R^2<0.7) (RS | WRS)	Prec. (R^2≥0.7) (RS | WRS)
NEE	Overall	4466 | 6849	59.5% | 83.4%	35.4% | 11.5%	5.1% | 5.1%
	Asia	1947 | 3422	41.7% | 59.0%	15.5% | 19.7%	42.8% | 21.3%
	Europe	2519 | 3427	63.4% | 94.2%	30.6% | 5.5%	6.0% | 0.3%
	Arid	1228 | 2148	58.6% | 48.6%	5.5% | 16.2%	35.9% | 35.2%
	Non-arid	3238 | 4701	53.2% | 89.5%	35.6% | 10.0%	11.2% | 0.5%
	Wetland	55 | 77	40.0% | 68.8%	5.5% | 9.1%	54.5% | 22.1%
	Cropland	1035 | 1294	72.9% | 42.4%	12.3% | 19.2%	14.8% | 38.4%
	Grassland	1996 | 2775	35.7% | 40.5%	30.3% | 23.9%	34.0% | 35.6%
	Forest	287 | 444	19.5% | 46.2%	49.8% | 39.2%	30.7% | 14.6%
	Total	4466 | 6849	15.5% | 31.8%	35.8% | 28.1%	48.7% | 40.1%
WF	Overall	4466 | 6849	10.2% | 22.4%	22.6% | 46.7%	67.2% | 30.9%
	Asia	1947 | 3422	25.9% | 42.5%	23.7% | 28.2%	50.4% | 29.3%
	Europe	2519 | 3427	3.8% | 9.5%	20.1% | 39.1%	76.1% | 51.4%
	Arid	1228 | 2148	18.8% | 26.1%	7.5% | 14.1%	73.7% | 59.8%
	Non-arid	3238 | 4701	8.0% | 10.7%	31.0% | 49.1%	61.0% | 40.2%
	Wetland	55 | 77	47.3% | 42.9%	16.3% | 5.2%	36.4% | 51.9%
	Cropland	1035 | 1294	23.3% | 24.3%	12.1% | 25.2%	64.6% | 50.5%
	Grassland	1996 | 2775	1.5% | 5.4%	5.4% | 14.6%	93.1% | 80.0%
	Forest	287 | 444	4.2% | 10.8%	25.1% | 22.8%	70.7% | 66.4%
	Total	4466 | 6849	0.9% | 1.3%	4.4% | 17.3%	94.7% | 81.4%

Prec., percentage of meteorological stations; N, number of meteorological stations; R^2, determination coefficient; Total, the maximum predicted R^2 for each meteorological station was selected for counting under 9 categories.

Table S6. Carbon-water flux datasets used for comparison with the results of this study.

Dataset	Variable	SR	TR	Data source
FLUXCOM	NEE	0.5°	Monthly	NEE.RF.CRUNCEPv6[8] (http://www.fluxcom.org/CF-Download/)
	LE	0.5°	Monthly	LE.RS_METEO.EBC-ALL.MLM-ALL.METEO-ALL.720_360[9] (http://www.fluxcom.org/EF-Download/)
GOSAT	BIOSPHERE FLUX	1°	Monthly	GOSAT L4A (https://data2.gosat.nies.go.jp/)
MODIS	LE	500m	8-Day	MOD16A2 Version 6[10] (https://doi.org/10.5067/MODIS/MOD16A2.006)

SR, spatial resolution; TR, temporal resolution; NEE, net ecosystem carbon dioxide exchange; LE, latent heat flux; BIOSPHERE FLUX, representing surface carbon flux in terrestrial ecosystems.

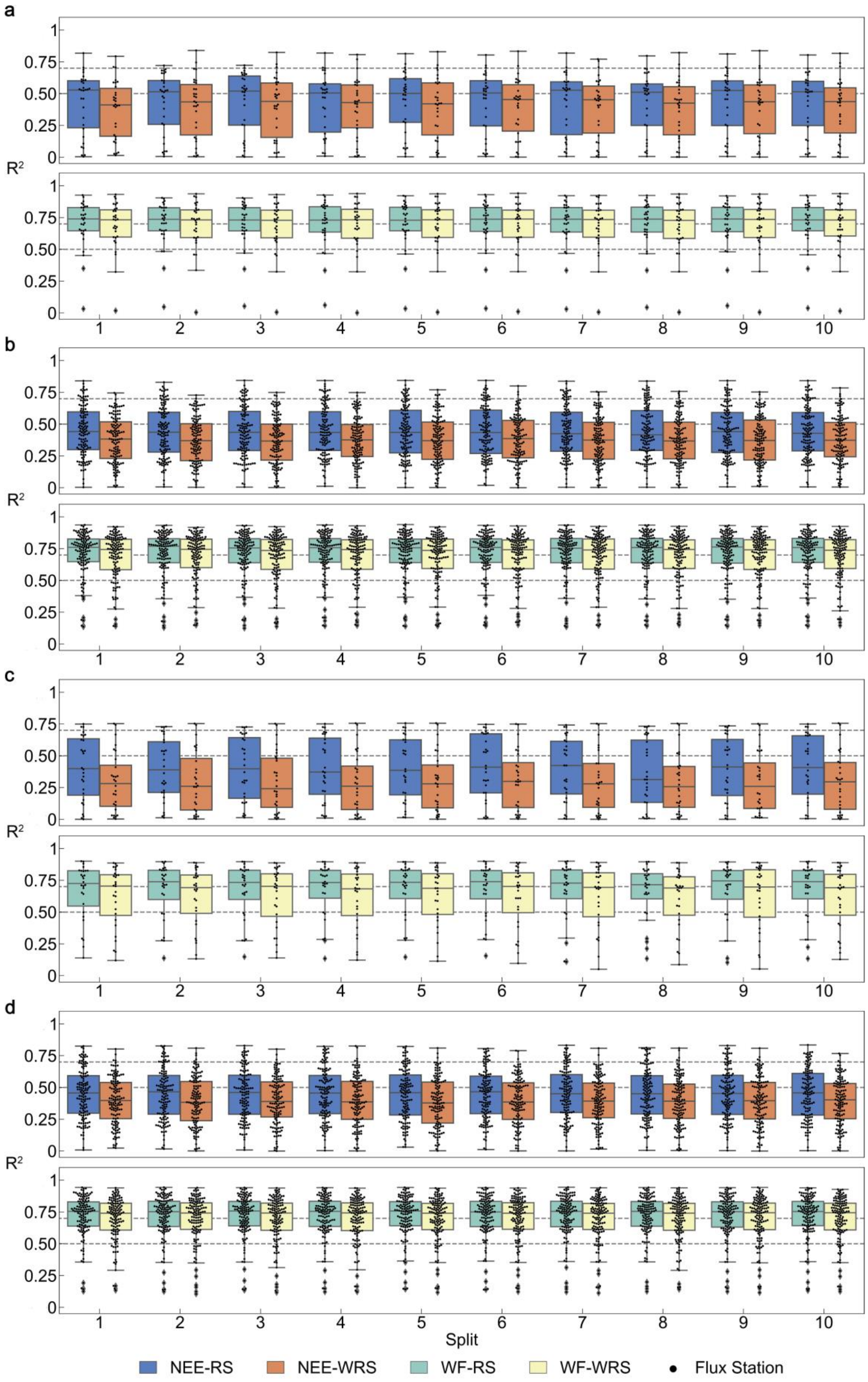

Fig. S1 The accuracy performance of the carbon-water flux simulation models (random forest

models, RFM) at test flux stations. NEE (net ecosystem carbon dioxide exchange) and WF (water flux) R^2-based accuracy performance of RFM in each split of the 10-time 10-fold cross-validation for (**a**) Asia with 30 stations, (**b**) Europe with 126 stations, (**c**) Arid with 28 stations and (**d**) Non-arid with 128 stations. Box plots show the R^2 distribution of each flux station of the test set for different categories, in which the whiskers indicate the 1.5 times' interquartile range.

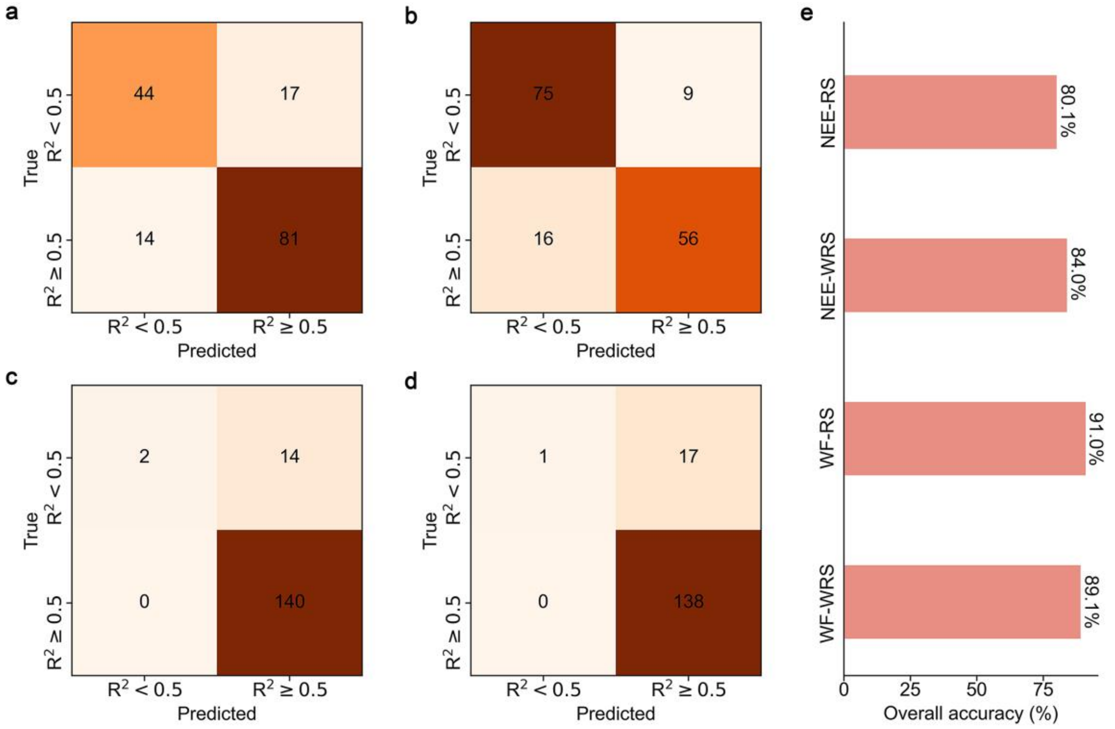

Fig. S2 Simulation accuracy of R^2 simulation model (RSM) at 156 test flux stations for (**a**) NEE-RS, (**b**) NEE-WRS, (**c**) WF-RS and (**d**) WF-WRS. Confusion matrixes showed the classification accuracy of RSMs through the true R^2 (tested by carbon-water flux simulation models) and predicted R^2 (predicted by the RSM) of test flux stations. (e) The overall accuracies of the RSM for a correct classification of R^2 for the NEE-RS, NEE-WRS, WF-RS and WF-WRS.

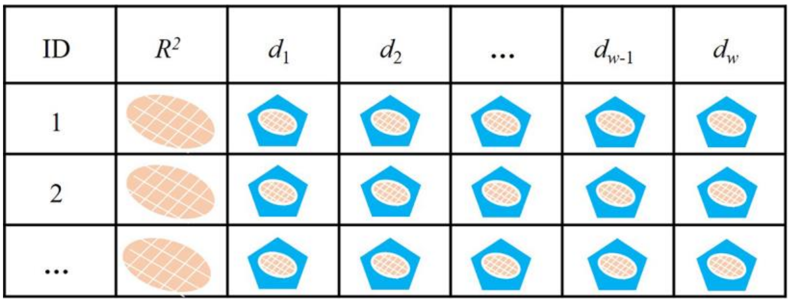

Fig. S3 Database structure of R^2 values of test flux stations and Euclidean distances of its influencing factors between test flux stations and training sets of RFM.

References

1. Tang, W. Dataset of high-resolution (3 hour, 10 km) global surface solar radiation (1983-2018). *National Tibetan Plateau Data Center* https://cstr.cn/18406.11.Meteoro.tpdc.270112 (2019).
2. Tang, W., Yang, K., Qin, J., Li, X. & Niu, X. A 16-year dataset (2000-2015) of high-resolution (3 h, 10 km) global surface solar radiation. *Earth Syst. Sci. Data* **11,** 1905–1915 (2019).
3. Liang, S.*, et al.* The global land surface satellite (GLASS) product suite. *Bull. Amer. Meteorol. Soc.* **102,** 1-37 (2020).
4. Myneni, R., Knyazikhin, Y. & Park, T. MCD15A3H MODIS/Terra+Aqua Leaf Area Index/FPAR 4-day L4 Global 500m SIN Grid V006. *NASA EOSDIS Land Processes DAAC* https://doi.org/10.5067/MODIS/MCD15A3H.006 (2015).
5. Vermote, E. & Wolfe, R. MOD09GA MODIS/Terra Surface Reflectance Daily L2G Global 1kmand 500m SIN Grid V006. *NASA EOSDIS Land Processes DAAC* https://doi.org/10.5067/MODIS/MOD09GA.006 (2015).
6. Yamazaki, D.*, et al.* A high-accuracy map of global terrain elevations. *Geophys. Res. Lett.* **44,** 5844–5853 (2017).
7. FAO/IIASA/ISRIC/ISSCAS/JRC. Harmonized World Soil Database (version 1.2). *FAO, Rome, Italy and IIASA, Laxenburg, Austria* https://webarchive.iiasa.ac.at/Research/LUC/External-World-soil-database/HTML (2012).
8. Jung, M.*, et al.* Scaling carbon fluxes from eddy covariance sites to globe: Synthesis and evaluation of the FLUXCOM approach. *Biogeosciences* **17,** 1343-1365 (2020).
9. Jung, M.*, et al.* The FLUXCOM ensemble of global land-atmosphere energy fluxes. *Sci. Data* **6,** 1-14 (2019).
10. Running, S., Mu, Q. & Zhao, M. MOD16A2 MODIS/Terra Net Evapotranspiration 8-Day L4 Global 500m SIN Grid V006. *NASA EOSDIS Land Processes DAAC* https://doi.org/10.5067/MODIS/MOD16A2.006 (2017).
